# Task modulation of spatiotemporal dynamics in semantic brain networks: An EEG/MEG study

**DOI:** 10.1016/j.neuroimage.2021.118768

**Published:** 2022-02-01

**Authors:** Setareh Rahimi, Seyedeh-Rezvan Farahibozorg, Rebecca Jackson, Olaf Hauk

**Affiliations:** a MRC Cognition and Brain Sciences Unit, University of Cambridge, 15 Chaucer Road, Cambridge CB2 7EF, United Kingdom; b Wellcome Centre for Integrative Neuroimaging, Nuffield Department of Neurosciences, University of Oxford, United Kingdom

**Keywords:** Semantic representation, Semantic control, Controlled semantic cognition, Source estimation, Leakage, MEG

## Abstract

How does brain activity in distributed semantic brain networks evolve over time, and how do these regions interact to retrieve the meaning of words? We compared spatiotemporal brain dynamics between visual lexical and semantic decision tasks (LD and SD), analysing whole-cortex evoked responses and spectral functional connectivity (coherence) in source-estimated electroencephalography and magnetoencephalography (EEG and MEG) recordings. Our evoked analysis revealed generally larger activation for SD compared to LD, starting in primary visual area (PVA) and angular gyrus (AG), followed by left posterior temporal cortex (PTC) and left anterior temporal lobe (ATL). The earliest activation effects in ATL were significantly left-lateralised. Our functional connectivity results showed significant connectivity between left and right ATL, PTC and right ATL in an early time window, as well as between left ATL and IFG in a later time window. The connectivity of AG was comparatively sparse. We quantified the limited spatial resolution of our source estimates via a leakage index for careful interpretation of our results. Our findings suggest that the different demands on semantic information retrieval in lexical and semantic decision tasks first modulate visual and attentional processes, then multimodal semantic information retrieval in the ATLs and finally control regions (PTC and IFG) in order to extract task-relevant semantic features for response selection. Whilst our evoked analysis suggests a dominance of left ATL for semantic processing, our functional connectivity analysis also revealed significant involvement of right ATL in the more demanding semantic task. Our findings demonstrate the complementarity of evoked and functional connectivity analysis, as well as the importance of dynamic information for both types of analyses.

## Highlights

• Semantic task demands affect activity and connectivity at different processing stages.
• Earliest task modulations occurred in posterior visual brain regions.
• ATL, PTC and IFG effects reflect task-relevant retrieval of multimodal information.
• ATL effects are left-lateralised for activation but bilateral for functional connectivity.
• Dynamic evoked and connectivity data are essential to study semantic networks.

## 1 Introduction

Semantics, or the representation and mental manipulation of our knowledge about objects, facts and people, is a crucial component of human cognition, underpinning all meaningful interactions with our environment and communication with others (Jefferies, 2013; Patterson et al., 2007). Our semantic system enables us to store, employ, manipulate, and generalise conceptual knowledge (Lambon Ralph et al., 2016). Learning and storing multimodal semantic representations are essential for successful semantic cognition, but they are not sufficient. The relevant information to deploy in any particular moment is context-sensitive and task-dependant, thus, we require semantic control to manipulate and shape the activation in the representation system (Jackson, 2021; Jefferies, 2013). However, task effects on brain dynamics during semantic processing are still largely unexplored.

The Controlled Semantic Cognition (CSC) framework proposes an interaction between control and representation regions in the brain, with semantic representation underpinned by a central semantic hub located in the anterior temporal lobes (ATL) (Lambon Ralph et al., 2016). Ample evidence for this proposal has been provided by studies on semantic dementia patients, who show specific semantic deficits following impairment of the anterior temporal lobes (Mion et al., 2010; Nestor et al., 2006), and fMRI and PET studies demonstrating ATL sensitivity to semantic stimulus and task manipulations (Crinion et al., 2003; Embleton et al., 2006; Mummery et al., 2000; Rogers et al., 2006; Tranel et al., 2005; Visser et al., 2012, 2010). Several studies have demonstrated similar effects in brain activity estimated from EEG or MEG data (Cope et al., 2020; Dhond et al., 2007; Farahibozorg et al., 2019; Marinkovic et al., 2014, 2003; Mollo et al., 2017), but the precise time course of semantic processing, as reflected in the brain activation or connectivity measures, has not been established yet. As a result, crucial evidence for the dynamic functional organisation of the semantic brain network is still missing, since temporal information is essential to disentangle effects that may occur at different stages of semantic processing, e.g., early semantic information retrieval, control processes in decision making, and later imagery or episodic memory processes (Hauk, 2016).

Temporal information is also particularly important for the reliable estimation of brain connectivity, since brain areas may play different roles at different stages of processing, and therefore dynamically change their connectivity. Furthermore, there is evidence that activity in different brain networks, arguably corresponding to different brain functions, are reflected in different frequency bands of electrical brain signals (Fries, 2015; Palva and Palva, 2012; Siegel et al., 2012). A number of studies have investigated the roles of different frequency bands in semantic processing (Bastiaansen et al., 2008; Mollo et al., 2017; Strauß et al., 2014; Teige et al., 2019; van Ackeren et al., 2014; van Ackeren and Rueschemeyer, 2014). In ATL, for example, increased theta power and decreased alpha and beta power have been associated with multimodal integration of lexical-semantic information in word (van Ackeren and Rueschemeyer, 2014) and object (Urooj et al., 2014) recognition, respectively. However, other studies (e.g. Mollo et al., 2017) have reported power reduction (semantic task vs baseline) in the gamma band at earlier time points, and across frequency bands at later time points. Thus, there is still no consensus on the specific roles of different frequency bands for semantics. As a result, we investigated functional connectivity in four different frequency bands (theta, alpha, beta, gamma) in our study.

Whilst ATL has consistently been linked to semantic representation (Acosta-Cabronero et al., 2011; Binder et al., 2016; Martin, 2016; Pobric et al., 2007; Rogers et al., 2004), IFG and pMTG are specifically implicated in semantic control (Badre et al., 2005; Jackson, 2021; Jefferies, 2013; Jefferies and Lambon Ralph, 2006; Lambon Ralph et al., 2016; Noonan et al., 2013). The role of AG is less clear and has been suggested to involve semantic representation (Binder et al., 2009), control (Noonan et al., 2013) or episodic memory processes (Humphreys et al., 2015). Thus, semantic cognition is dependant on semantic representation in the ATL and sensory-specific regions, and control in IFG and pMTG, with a possible role for the AG. Few studies have investigated the interaction between semantic control and representation regions, and the connectivity and temporal dynamics of the corresponding brain regions are still not well understood (Jefferies, 2013; Lambon Ralph et al., 2016). Most previous studies of the semantic network and its connectivity have employed fMRI (Alam et al., 2021; Chiou et al., 2018; Chiou and Lambon Ralph, 2019; Humphreys et al., 2015; Jackson et al., 2016; Kuhnke et al., 2020) which despite its excellent spatial resolution, is limited in tracking any neural response faster than one second.

In the present study, we provide novel evidence for task effects on the spatiotemporal dynamics in the semantic network and, in particular, functional connectivity amongst these regions. EEG and MEG are sensitive to semantic stimulus manipulations in different time windows, such as the N400 latency range (typically between 250 and 500 ms) (Kutas and Federmeier, 2011; Lau et al., 2008) and earlier (Amsel et al., 2013; Hauk et al., 2012; Pulvermüller et al., 2009). Importantly, source estimation with MEG has revealed lexicosemantic effects in the anterior and middle temporal lobes (Dhond et al., 2007; Farahibozorg et al., 2019; Flick et al., 2018; Hauk et al., 2012; Lau et al., 2013; Mollo et al., 2017), inferior parietal cortex (Bemis and Pylkkänen, 2013; Farahibozorg et al., 2019; Lewis et al., 2015; Williams et al., 2017), and inferior frontal cortex (Schoffelen et al., 2017; Woodhead et al., 2014). Furthermore, semantic task manipulations have been reported to modulate EEG/MEG signals in early and late time windows (Chen et al., 2015; Chen et al., 2013) and in the frequency domain (Clarke et al., 2011; Lewis and Bastiaansen, 2015; Mollo et al., 2017).

Here, we investigated the effects of different semantic task demands on dynamic brain activity and spectral functional connectivity in the semantic brain networks. We used a whole-cortex approach initially, but also focused on prominent regions-of-interest (ROIs) that have previously been implicated in semantic representation and control, as described above: ATL, IFG, pMTG, and AG. Most previous studies have found the semantic brain network to be left-lateralised (Binder et al., 2009). Yet, a notable exception is the ATL, for which a graded lateralisation has been reported depending on stimulus and task features (Lambon Ralph et al., 2010; Marinkovic et al., 2003; Olson et al., 2007; Patterson et al., 2007; Pobric et al., 2007; Rice et al., 2015b, 2015a; Visser et al., 2010). Thus, our ROIs will include both left and right ATL to study the laterality of task effects in this region.

We contrasted brain dynamics between two visual word recognition tasks, namely lexical and semantic decisions on the identical word stimuli. In the lexical decision (LD) task, participants had to distinguish between words and pseudowords. This task only explicitly requires the classification of letter strings as existing words or not, and therefore does not explicitly demand the retrieval of specific semantic features. However, the harder the distinction between the words and pseudowords, the more these decisions are affected by semantic variables, and lexical decision is compromised with impaired semantic representations (Evans et al., 2012; Patterson et al., 2006). This task is therefore suitable to evoke activity in the semantic network. We compared this task with a semantic decision (SD) task which explicitly required participants to retrieve specific semantic information about the words (such as “Is it something edible with a distinctive odour?”). This ‘task differences’ approach (Chen et al., 2015; Chen et al., 2013; Kuhnke et al., 2021, 2020) employs a high-level baseline, providing a powerful way to identify specific changes with greater semantic processing, such as the particular timing of differences. By presenting the same stimuli in two different tasks we can assess the effect of demanding semantic processing over and above the effect of presenting meaningful stimuli.

We used spectral coherence as a functional connectivity metric, as it is sensitive to covariations of both phase and amplitude across trials between signals from two regions (Bastos and Schoffelen, 2016). We investigated the potential effect of source leakage in an explicit resolution analysis of our measurement configuration (Hauk et al., 2019). Specifically, we asked 1) how task modulation of semantic brain activity evolves across time, 2) how connectivity of putative semantic representation and control regions is affected by task demands over time, and 3) how task demands modulate the laterality and connectivity of left and right ATLs.^1^

## 2 Materials and methods

### 2.1 EEG/MEG experiment data acquisition

#### 2.1.1 Participants

26 healthy native adult English speakers (age 18–40) participated, 2 of whom were excluded due to problems with structural MRI scans. 3 were excluded due to inadequate behavioural response accuracies (less than 75% response accuracy) and 3 were excluded because of excessive movement artefacts. The excessive movement artefacts were determined based on: visual inspection by two authors, number of bad channels and number of bad epochs. Therefore, 18 participants (mean age 27.00±5.13, 12 female) entered the final analysis. A reduced version of the Oldfield handedness inventory (Oldfield 1971) was used, based on which a mean handedness laterality quotient of 89.84±0.2 was obtained. All participants had normal or corrected-to normal vision and reported no history of neurological disorders or dyslexia. The experiment was approved by the Cambridge Psychology Research Ethics Committee and volunteers were paid for their time and effort. (This experiment and its full details are described in Farahibozorg, 2018)

#### 2.1.2 Stimuli

The stimulus set included in our MEG analysis consisted of 250 uninflected words, including three categories of concrete words with strong visual, auditory and hand-action attributes (50 words per category), as well as two categories of emotional and neutral abstract words (50 words per category). For the purpose of this study, all the 250 words were pooled and a summary of their psycholinguistic variables as well as those of pseudowords are presented in Table 1. Concreteness ratings were obtained based on a word rating study (Farahibozorg, 2018) and CELEX Frequency, Orthographic Neighbourhood, Bigram and Trigram Frequencies were taken from the MCWord Database (Medler and Binder, 2005). Additional filler pseudowords were also included in the experiment, which are not assessed in this study.

**Table 1:** Psycholinguistic properties of stimuli included in EEG/MEG data analysis. The same words were used in the LD and SD tasks.

average ± standard deviation		
	Words	Pseudowords
Number of Letters	5.68±1.56	5.0 ± 1.0
CELEX Frequency	16.13±22.14	N/A
Orth Neighbourhood	3.78±4.81	4.70±4.50
Bigram Frequency	19,008.54±9584.31	19,465.83±10,435.78
Trigram Frequency	1866.29±2278.84	1670.66±2029.80
Concreteness Rating	4.44±1.72	N/A

#### 2.1.3 Procedure

The EEG/MEG experiment comprised four blocks presented in random order, and lasted approximately 90 min. We included 10-minute breaks between the blocks and short breaks every three minutes within each block. Each stimulus was presented for 150 ms, with an average SOA of 2400 ms (uniformly jittered between 2150 and 2650 ms). Stimuli appeared as 30-point Arial font in black on a grey screen within a visual angle of 4° in a slightly dimmed and acoustically shielded MEG chamber. One of the four blocks consisted of a lexical decision task and the remaining three blocks consisted of semantic target detection tasks. Half of the participants were randomly assigned to perform the lexical decision first and the other half performed semantic target detection blocks first. Details of these blocks were as follows:

1) Semantic target detection blocks: In each block, participants were presented with 250 words, as well as the filler items (overall 300 stimuli), in addition to 30 targets. They were asked to quietly read the strings of letters as they appeared on the screen and make button press responses with their left-hand middle finger only when they saw a target (30 words per each SD block) on the screen. Each block had different targets which were selected from three groups of “non-citrus fruits”, “something edible with a distinctive odour” and “food that contains milk, flour or egg”. Participants were required to choose their responses with respect to the same question for every trial within a block. Block orders were randomised across participants, and data acquired from the three blocks were pooled in the later EEG/MEG analyses to avoid possible question-specific effects.
2) Lexical decision task: Participants also performed a lexical decision task with the same 250 words, and 250 filler pseudowords to acquire response balance across stimuli (overall 500 stimuli). Participants were asked if “the following string of letters refers to a meaningful word” and they were asked to make button press responses with the index and ring fingers of their left hand for words and pseudowords, respectively. Only word stimuli were included in the subsequent EEG/MEG analyses.

**Fig. 1:**
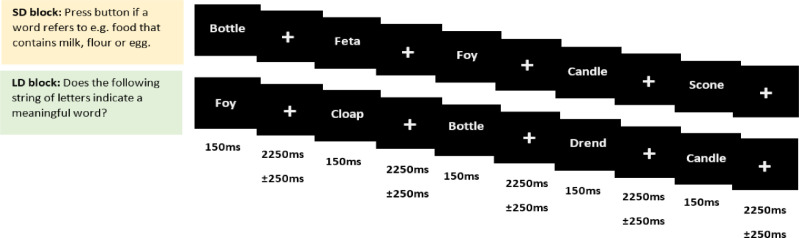
Illustration of trials and timings of semantic decision and lexical decision tasks. Each word is presented for 150 ms, followed by a 2250±250 ms gap. The SD task consisted of three separate blocks with three different questions while the LD task consisted of one block.

The SD task was chosen to strongly engage participants in deep semantic processing through the need to access specific semantic features to select an appropriate response, in contrast to the LD task which does not explicitly require the retrieval of semantic information for response selection but has been shown to engage semantic processing to some degree (Evans et al., 2012). Fig. 1 shows the format of the task and its timings. All participants could do both tasks with high accuracy in the pilot and the main study (SD block accuracy: 0.90±0.11%, LD block accuracy: 95.09±3.96%). The choice of tasks led to differences in response type to words, i.e. no responses to words in SD (except in catch trials) and responses to all words (and pseudowords) in LD. This choice was mainly based on pragmatic considerations, since a two-alternative forced choice design is standard for LD and provides behavioural data for words, while in our SD task it would have been near-impossible to design a stimulus set with equal numbers of yes/no responses (comparable to LD) and requiring 90% of No responses in addition to the 10% catch trials would have been unconventional and possibly confusing for our participants. We do not consider the details of response execution at the end of each trial as a serious confound for our EEG/MEG results in earlier latency ranges. Average response time to words in the LD task was 660 ms. Even allowing for some variability across trials, our results are unlikely to have been affected by response selection (see also Hauk et al., 2012). For example, we would not be able to explain differential involvement of ATL and AG in semantic networks depending on response type (rather than semantic task demands). If this was the case, it would throw serious doubt on the ecological validity of results from any study using laboratory tasks such as LD and SD, which with respect to response type are arguably more different from natural reading than from each other. This should be further investigated in future studies (Hauk and Weiss, 2020).

### 2.2 Data acquisition and pre-processing

MEG and EEG data were acquired simultaneously using a Neuromag Vectorview system (Elekta AB, Stockholm, Sweden) and MEG-compatible EEG cap (EasyCap GmbH, Herrsching, Germany) at the MRC Cognition and Brain Sciences Unit, University of Cambridge, UK (Farahibozorg, 2018). MEG was recorded using a 306-channel system that comprised 204 planar gradiometers and 102 magnetometers. EEG was acquired using a 70-electrode system with an extended 10–10% electrode layout. EEG reference and ground electrodes were attached to the left side of the nose and the left cheek, respectively. ElectroOculoGram (EOG) was recorded by placing electrodes below and above the left eye (vertical EOG) and at the outer canthi (horizontal EOG). Electrocardiogram (ECG) was recorded by placing one electrode on the lower left rib and another electrode on the right wrist. Data were acquired with a sampling rate of 1000 Hz and an online band-pass filter of 0.03 to 330 Hz. During pre-acquisition preparations, positions of 5 Head Position Indicator (HPI) coils attached to the EEG cap, 3 anatomical landmark points (two ears and the nose) as well as approximately 50–100 additional points that covered most of the scalp were digitised using a 3Space Isotrak II System (Polhemus, Colchester, Vermont, USA) and later used for co-registration of EEG/MEG recordings with MRI data.

We applied signal space separation with its spatiotemporal extension implemented in the Neuromag Maxwell-Filter software to the raw MEG data to remove noise generated from sources distant to the sensor array (Taulu and Kajola, 2005). All remaining analyses were performed in the MNE-Python software package (Gramfort et al., 2014; A. 2013). Raw data were visually inspected for each participant, and bad EEG channels were marked and linearly interpolated. Data were then band-pass filtered using a finite-impulse-response (FIR) filter between 0.1 and 45 Hz. FastICA algorithm (Hyvarinen, 1999; Hyvärinen and Oja, 2000) was applied to the filtered data to remove eye movement and heartbeat artefacts. After ICA, data were divided into epochs from 300 ms pre-stimulus to 600 ms post-stimulus.

### 2.3 Source estimation

We used L2-Minimum Norm Estimation (MNE) (Hämäläinen and Ilmoniemi, 1994; Hauk, 2004) for source reconstruction. Inverse operators were assembled based on a 3-layer Boundary Element Model (BEM) of the head geometry derived from structural MRI images, assuming sources perpendicular to the cortical surface (“fixed” orientation constraint). The MEG sensor configurations and MRI images were co-registered by matching the scalp digitisation points from the MEG preparation to the scalp surface reconstructed from individual MRI images. The noise covariance matrices for each individual and run were calculated for baseline intervals of 300 ms. To do so, we used a list of methods from MNE python, 'shrunk', 'diagonal_fixed', 'empirical', 'factor_analysis', and the best estimator (’shrunk’ in most cases) was selected using log-likelihood and cross-validation (Engemann and Gramfort, 2015). MNE-Python's default SNR = 3.0 was used for evoked responses to regularise the inverse operator. The individuals’ results were then morphed to the standard average brain (fsaverage), yielding the time courses of activity for 20.484 vertices for each subject and condition. It is noteworthy that the non-uniqueness of the EEG/MEG inverse problem leads to restricted spatial resolution, which may result in systematic mislocalisation of the genuine sources (Fuchs et al., 1999; Hauk et al., 2011; Molins et al., 2008), or more generally signal leakage between regions (Colclough et al., 2015; Palva et al., 2018; Wens et al., 2015; Williams et al., 2019).

### 2.4 Regions of interest

**Fig. 2:**
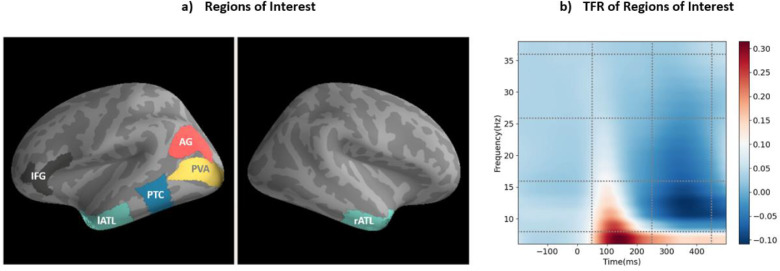
a) Regions of interest (ROIs) based on the semantic literature, b) Time-Frequency Representation (TFR) across all ROIs, tasks, and participants.

Six regions of interest were defined using the anatomical masks provided from the Human Connectome Project (HCP) parcellation (Glasser et al., 2016), to represent the core semantic network as described in the introduction. As Fig. 2a shows, this includes left and right ATL (as defined in HCP: TGd, TGv, TE1a, anterior portions of TE2a and TE1m cut to terminate at the posterior extent of TE1a), left IFG (44, 45, 47l, p47r), left posterior temporal cortex (PTC, including posterior middle and inferior temporal gyri) (TE1p and posterior portions of STSvp, anterior inferior part of pH, and posterior portion of TE2p, all cut to terminate at the anterior limit of TE1p), left AG (PGi, PGp, PGs) and left primary visual area (PVA) (V1, V2, V3, V4).

### 2.5 Leakage

Source leakage is inherent in EEG/MEG source estimation due to the non-uniqueness of the inverse problem. Here, we provide a quantitative description of the source leakage amongst our ROIs. To have a better insight into the pattern of potential leakage, we computed the point spread and cross-talk functions (PSFs and CTFs; Hauk et al., 2011; Liu et al., 2002) of all the ROIs, to test how activity from one ROI leaks or spreads out to other regions or the other way around. The general idea is to estimate the leakage from each ROI into all ROIs, relative to each ROI's leakage into itself, to generate an ROI-to-ROI leakage matrix.

Thus, we defined the leakage index (LI) as follows:

$$LI{\;}_{ij}=\frac{L_{ij}\;}{L_{jj}\;}$$

Where $L_{ij}$ is leakage from $ROI_{i}$ into $ROI_{j}$ and $L_{jj}$ is leakage from $ROI_{j}$ into itself. Leakage can be described by PSFs, i.e., how each ROI leaks into the other ROIs, and CTFs, i.e., how all ROIs leak into one particular ROI. For the unweighted L2 minimum norm estimate, PSFs and CTFs are the same (its resolution matrix is symmetric, Hauk et al., 2019), and the leakage matrix, therefore, represents both types of leakage (similar to Farahibozorg et al., 2018).

**Fig. 3:**
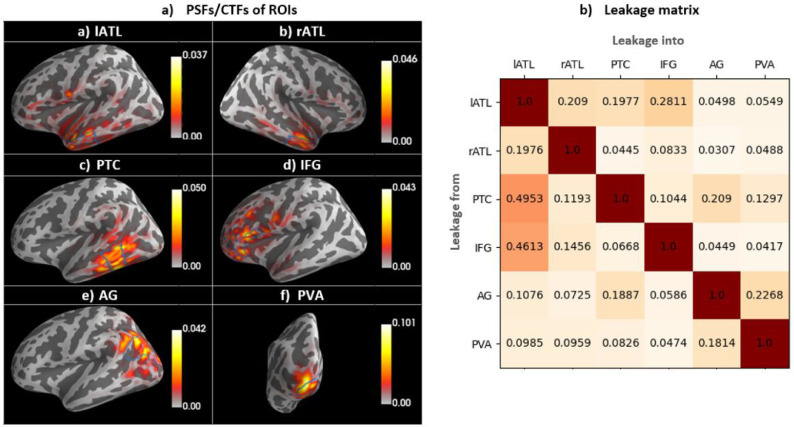
Leakage patterns. a) Grand average of PSFs/CTFs for each ROI, indicating how a real point source would leak to other regions (PSFs)/how all regions would leak to a particular ROI (CTFs). ROI borders are specified by solid blue lines. b) the leakage matrix; every column, corresponding to a single ROI, shows how much other regions leak into that ROI relative to what it leaks into itself.

Fig. 3 presents PSFs and CTFs for our ROIs, as well as their associated leakage matrix. This shows that leakage varies across pairs of ROIs. To describe this variability, we will consider leakage indices between 0-0.2/0.2–0.4/0.4–0.6/0.6–0.8/0.8–1 as low/low-medium/medium/medium-high/high, which is reflected in the shading of the matrix cells. Leakage was medium and lower across all pairs of ROIs, and all leakage indices were below 0.5. Medium and high amounts of leakage can indicate that connectivity obtained from a pair of ROIs will be more affected by the limitations of the spatial resolution of the EEG/MEG source localisation and, thus, should be interpreted with more caution. Fig. 3a confirmed that our ROIs produced most leakage in their vicinity. We will take individual leakage indices into account in our interpretation and discussion where appropriate. The PSFs/CTFs suggest that some ROIs have a wider distribution than others (e.g., PTC vs. ATL). This could be related to different factors, including the geometry of the ROIs, size of the ROIs, distance from the sensors, source depth, and source orientation, etc. (Hauk et al., 2019), which should be explored further in future studies. Please note that it is currently uncommon for non-methodological EEG/MEG studies to report this kind of information.

### 2.6 Evoked responses

The relevant trials for word stimuli were averaged in sensor space to obtain an evoked response per participant and task. Evoked responses were projected onto source space using L2-MNE (see above) and compared between the lexical and semantic decision tasks. For statistical analysis of the whole-cortex evoked responses, we used spatiotemporal cluster-based permutation tests, mne.stats.spatio_temporal_cluster_1samp_test function implemented in MNE python, (Maris and Oostenveld, 2007), accounting for multiple observations across vertices and time points. For this purpose, t-values were computed and thresholded with a t-value equivalent to p-value < 0.05 for a given number of observations, and randomisation was replicated 5000 times to obtain the largest random clusters. The critical alpha-level for both vertex-wise and cluster-wise t-tests was 0.05. We applied two-sided t-tests and the upper (SD>LD) and lower (LD>SD) 0.025 in the resulting permutation distribution were considered to be significant. In addition to the whole cortex analyses, activation time-courses were extracted from each ROI (using MNE Python's “mean flip” option to account for varying source orientations within an ROI) and compared using cluster-based permutation tests per ROI.

As argued in (Sassenhagen and Draschkow, 2019), cluster-based permutation tests do not allow inferences about the spatial or temporal extent of clusters. However, testing for multiple latency ranges or samples poses a multiple comparisons problem. Here, we applied cluster-based permutation tests across vertices and samples in several latency windows for which lexico-semantic effects for stimulus and task features have been reported previously, namely two early windows around 100 ms (Chen et al., 2015; Hauk et al., 2006; Strijkers et al., 2015) and 200 ms (Amsel et al., 2013; Hauk et al., 2012; Pylkkänen, 2020), as well as the post-250-ms N400 period broken down into three time windows (Grainger and Holcomb, 2009; Kutas and Federmeier, 2011; Lau et al., 2008)). For the functional connectivity analysis we reduced this to two latency windows in order to increase frequency resolution. We tested independent hypotheses in each time window, and therefore did not correct for multiple comparisons across latency ranges. For the same reasons we did not apply correction for multiple comparisons across frequency bands in our functional connectivity analysis. Importantly, our conclusions are not based on the presence or absence of isolated effects, but on the pattern or results across several different analyses, such as which brain areas appear in most of our significant results.

### 2.7 Connectivity analyses

Functional connectivity was estimated based on spectral coherence because it is sensitive to covariations of both phase and amplitude between two signals (Bastos and Schoffelen, 2016). We were also interested in potential zero-lag connectivity (e.g., between left and right ATLs), and therefore did not use the imaginary part of coherency or signal orthogonalisation (Colclough et al., 2015; Nolte et al., 2004). Coherence measures the stability of the polar vectors (representing phase and amplitude at a specific frequency in a specific latency window in one trial) across trials (Bastos and Schoffelen, 2016). We will discuss any issues related to spatial resolution and leakage on the basis of our leakage analysis described above.

Whole-cortex seed-based connectivity was computed from each ROI. For this purpose, the ROI time-courses were extracted from each of the three blocks in the SD task, and from the LD task block. Magnitude-squared coherence was computed between each ROI time course and every vertex in the brain, for four different frequency bands and two time windows. The connectivity results were averaged across the three SD blocks for comparison with LD. This helped ensure that our coherence estimation is not biased due to different numbers of trials between the SD and LD task (Bastos and Schoffelen, 2016). This is equivalent to comparing each SD block with LD separately, and then averaging the three comparisons to reduce bias and variability across blocks. To choose frequency bands and time intervals of interest in an unbiased manner, we present the time-frequency representation of our dataset across all conditions, participants, and ROIs in Fig. 2b. Based on prominent features of this time-frequency representation, i.e., peaks, increases and decreases of activity, we selected an early (50–250 ms) and late (250–500 ms) time window and computed coherence in four frequency ranges, namely theta (4–8 Hz), alpha (8–16 Hz), beta (16–26 Hz), and gamma (26–36 Hz). Statistical comparisons for seed-based analyses (SD vs LD) were performed using cluster-based permutations, as described above. For between-ROIs analyses, we compared coherence values with paired two-sided t-tests.

## 3 Results

### 3.1 EEG/MEG behavioural results

For the lexical decision task, the average and standard deviation reaction times were 660±69 ms and response accuracies were 95.09±3.96%. For the semantic target detection blocks, the target detection accuracy and reaction times were 0.90±0.11 and 990±220 ms, respectively.

### 3.2 Whole-cortex evoked analysis

Most previous investigations into the neuronal basis of semantics using EEG/MEG and source estimation based their main conclusions on ROI-based analysis approaches. While this increases statistical sensitivity, it raises questions with respect to the spatial specificity of the reported effects, especially since the limited spatial resolution and possible mislocalisation of EEG/MEG source estimation are well-documented (Hauk et al., 2019; Molins et al., 2008). However, whole-cortex analyses in different latency and frequency ranges can be hard to present and interpret. In the following, we will present a hybrid approach that starts with whole-cortex results followed by ROI-based results. Our main conclusions will be based on the commonalities of the two analyses, and we will discuss any discrepancies where appropriate.

**Fig. 4:**
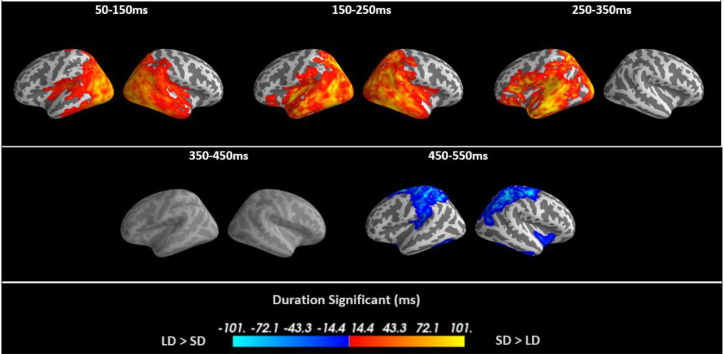
Spatiotemporal cluster-based permutation test contrasting the evoked responses of Semantic Decision (SD) and Lexical Decision (LD) in five time windows. The first three time windows showed significantly greater activation for SD than LD (hot colours), across the semantic network, with early effects in occipital and temporal cortex and later differences in frontal cortex. These changes are initially bilateral and later left-lateralised. The last time window demonstrates significant activation for LD (blue colours). The critical alpha-level used to threshold both vertex-wise and cluster-wise t-tests is 0.05. All coloured areas in the first two and the last time windows have p-values < 0.001 and all significant results in the third time window have p-values < 0.01.

To track task modulation of brain activation over time, we first compared evoked brain activity between our two tasks using whole-cortex cluster-based permutation tests in five non-overlapping time windows of 100 ms duration starting at 50 ms after stimulus onset. These brain dynamics were then analysed in more detail using an ROI analysis. The results of the whole-cortex evoked analysis are displayed in Fig. 4. The colour-coding indicates the duration of significant activation within each time window. Importantly, task differences were already apparent in the first time window (50–150 ms) and remained significant throughout the first three windows until 350 ms.

The semantic decision task produced higher levels of activation compared to the lexical decision task up to 350 ms. The earliest task differences were predominantly in bilateral posterior brain areas, but differences were already present in inferior parietal and anterior temporal brain regions. Between 150–250 ms, task modulations spread further into anterior temporal and parietal regions in both hemispheres. After 250 ms, activation was strongly left-lateralised and included left inferior frontal regions. There were no significant task differences between 350 and 450 ms. The lexical decision task produced larger activation than the semantic decision task between 450 and 550 ms. At this late latency, this could be due to late semantic processing partly overlapping with response planning and execution in the N400 time window, suppression of activation due to the absence of button presses to words in the SD task, and possible leakage from peak activation due to button presses in the right hemisphere.

As explained in 2.6, we did not consider it necessary to correct for multiple comparisons across latency windows, as our main conclusions are based on the pattern of results across several analyses. However, the significant effects of our whole-cortex evoked responses in Fig. 4 would survive Bonferroni correction across five time windows (uncorrected: p-values< [0.001, 0.001, 0.01, 0.8, 0.001]), FDR^2^ corrected p-values<[0.0016, 0.0016, 0.0125, 0.8, 0.0016], and Bonferroni^3^ corrected p-values<[0.005, 0.005, 0.05, 1, 0.005]).

### 3.3 ROI activation time-courses

**Fig. 5:**
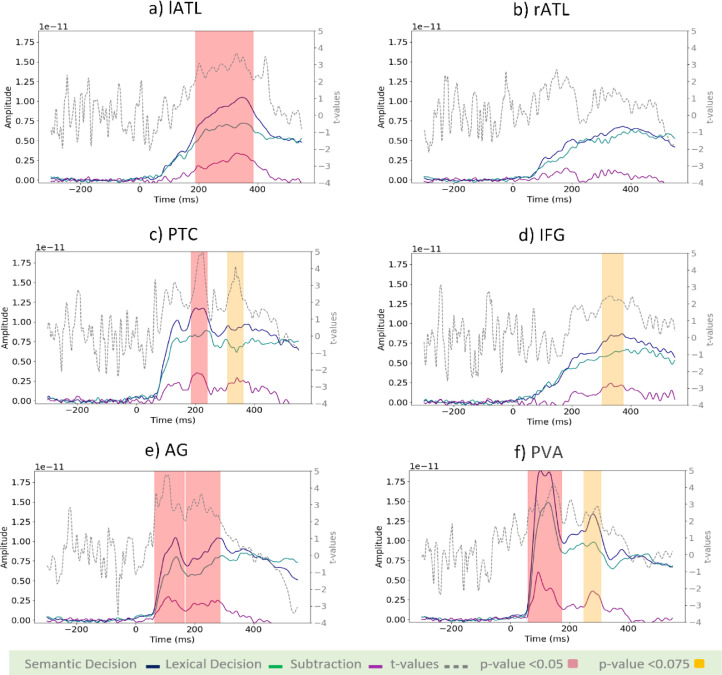
Activation time-course of ROIs for SD (blue lines), LD (green lines), SD-LD (purple lines), and t-values (dotted-grey lines). The left-hand side axis represents source amplitudes, the right-hand side axis shows t-values for the comparison of SD and LD, and the horizontal axis represents time in milliseconds. t-values corresponding to p-value <0.05 have been highlighted in red, and those with p-value <0.075 in yellow.

Fig. 5 presents the millisecond-by-millisecond time courses of evoked brain activity for our selection of ROIs. Averaged time courses across participants are shown for each individual task and their subtraction, alongside the t-values of their statistical comparison. Shaded areas highlight the latency ranges with significant task differences using cluster-based permutation tests. The earliest task differences occurred in PVA (p-value<0.05) and AG (p-value<0.01) from 60 to 65 ms. Note that the leakage indices for these two regions (Fig. 3b) were about 0.2, and their time courses are similar (Fig. 5e and f). Therefore, we cannot rule out the possibility that these results reflect leakage effects, i.e., are due to the same neuronal sources in posterior brain areas. These early effects were followed by differences in PTC (p-value<0.05) and lATL (p-value<0.01) at 186 and 189 ms. We also found marginally significant task differences at later latencies in PVA (p-value<0.075) at 300 ms, and IFG (p-value<0.075), and PTC (p-value<0.075) at 309 ms. We observed no statistically significant task difference in rATL at any latency.

### 3.4 ATL laterality

**Fig. 6:**
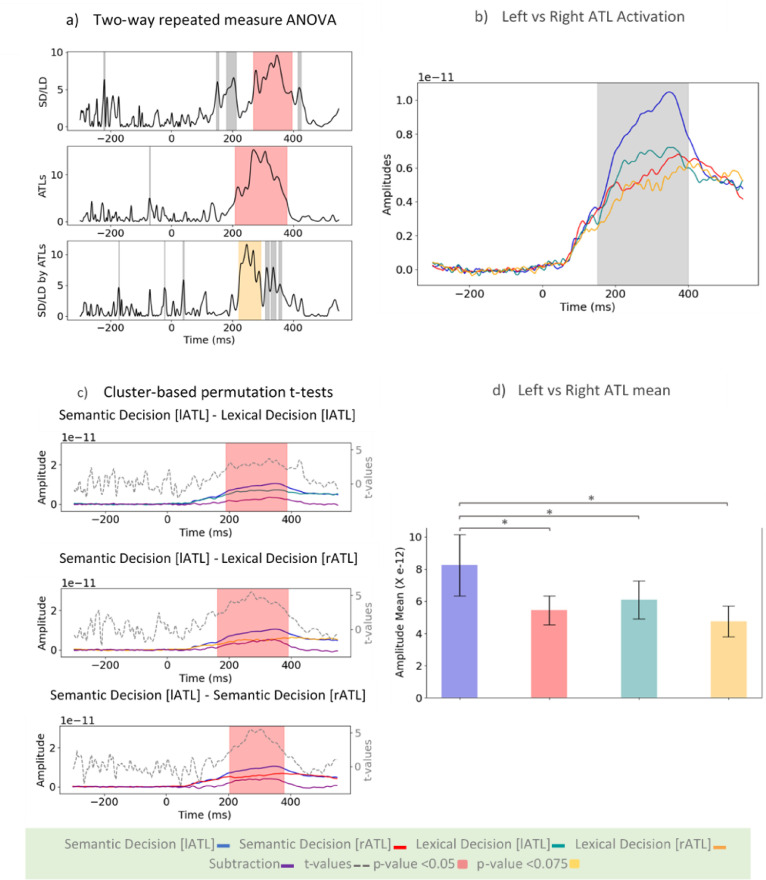
Laterality of task modulation in ATLs. a) the effect of Task (SD vs. LD), Laterality (left vs. right), and their interaction using a two-way repeated-measures ANOVA. b) activations of the left and right ATLs in SD and LD. c) all comparisons between the left and right ATL activation in each task that reach significance using cluster-based permutation test (three out of six). t-values corresponding to p-values <0.05 have been highlighted in red, and those with p-values <0.075 in yellow. d) average activation of the left and right ATLs in SD and LD tasks in the time range of 150 to 400 ms (shaded grey area in panel b), chosen based on the interaction results.

We explicitly tested the laterality of ATL involvement. Fig. 6a shows the main effects of task, laterality, and their interaction using a two-way repeated-measures ANOVA. To understand this interaction, six planned comparisons were run. Fig. 6b shows separate activation time courses for left and right ATL for lexical and semantic decision tasks, respectively. Fig. 6c displays the contrasts that yielded significant results. Fig. 6d presents a summary statistical analysis of activation averaged in the time window 150–400 ms. This analysis demonstrated that the task effects in the ATLs were driven by larger activation in the left, but not right, ATL for the semantic decision task than the lexical decision task ([SD[*l*ATL]-SD[*r*ATL]: (*t*=4.13, *p*<0.001)], [SD[*l*ATL]-LD[*l*ATL]: (*t*=3.00, *p*<0.01)], [SD[*l*ATL]-LD[*r*ATL]: (*t*=4.76, *p*<0.001)], [SD[*r*ATL]-LD[*l*ATL]: (*t*=−0.66, *p*>0.50)], [SD[*r*ATL]-LD[*r*ATL]: (*t*=1.24, *p*> 0.20)], [LD[*l*ATL]- LD[*r*ATL]: (*t*=1.82, *p*>0.08)]). Thus, the left and right ATL responded similarly to the less demanding lexical decision task, yet the increased requirements of the semantic decision task were met by a greater response from the left ATL in particular.

### 3.5 Connectivity analysis: whole-cortex seed-based connectivity

**Fig. 7:**
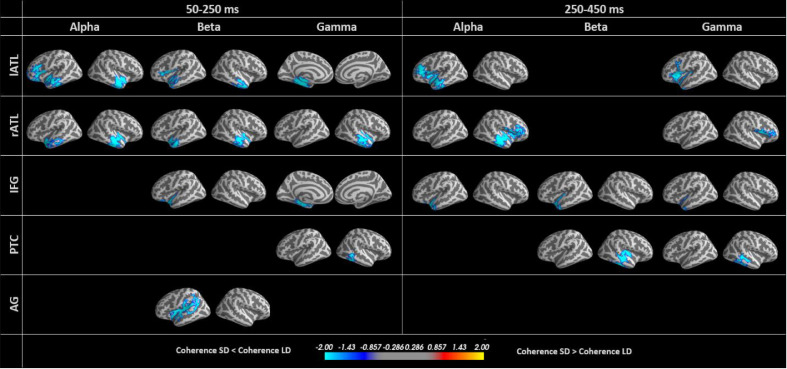
Whole-cortex seed-based connectivity differences between the semantic and lexical decision tasks for different ROIs, frequency and latency ranges. All coloured areas have p-values < 0.05. Blue colours show greater coherence for LD.

We studied task modulation of functional connectivity in the semantic network with a whole-cortex seed-based analysis, followed by ROI analyses. The seed-based analysis determined the coherence between our ROIs and all other vertices in the brain in two time windows (early 50–250 ms and late 250–450 ms) and four frequency bands (theta, alpha, beta, gamma). The whole-cortex seed-based connectivity results are presented in Fig. 7. Statistical significance was assessed based on whole-cortex cluster-based permutation tests. We found no significant effects in the theta band, which may be too slow to reflect the short-lived processes involved in semantic single-word processing.

The ROI labels in the left column indicate the seed region. Note that each seed region strongly “leaks” into itself (Fig. 3b), and therefore we can expect high coherence values within each seed region for the individual tasks. However, if these values are similar for lexical and semantic decisions, they will not produce significant effects in the subtraction (or statistical comparison). Significant effects in seed regions may still occur due to other factors (e.g., noise levels), but we will not interpret them in terms of functional connectivity. We will take possible leakage into account in the interpretation of functional connectivity (see Fig. 3).

Interestingly, our functional connectivity analyses generally revealed larger coherence values for lexical compared to semantic decisions, which may appear counterintuitive or in contrast to our evoked analyses showing larger activation for semantic decisions. However, this could be explained by larger trial-by-trial variability and higher desynchronisation leading to lower coherence in the more demanding task. We will come back to this issue in the discussion.

In the early time window, we observed significant task differences in functional connectivity between the left and right ATL in alpha and beta bands, as well as from AG along the Sylvian fissure, including anterior superior temporal lobe. The gamma band demonstrated significant modulation of connectivity between the IFG seed and left ATL, and between PTC and an approximately homologous area in the right hemisphere.

In the late time window, we found significant task modulation of connectivity between lATL and left IFG, as well as rATL and right IFG, but not between lATL and rATL as found in the early time window. AG did not demonstrate any connectivity differences in this time window, while PTC showed differential connectivity with an area of right middle temporal lobe in the beta and gamma bands. In the gamma band, there was task modulation of the connectivity between the lATL and IFG.Thus, most differences in the connectivity of the lexical and semantic decision tasks involved the ATLs, with task modulation principally affecting the connectivity between left and right ATL at early stages, and later, between IFG and ATL.

### 3.6 Connectivity analysis: between-ROIs connectivity

**Fig. 8:**
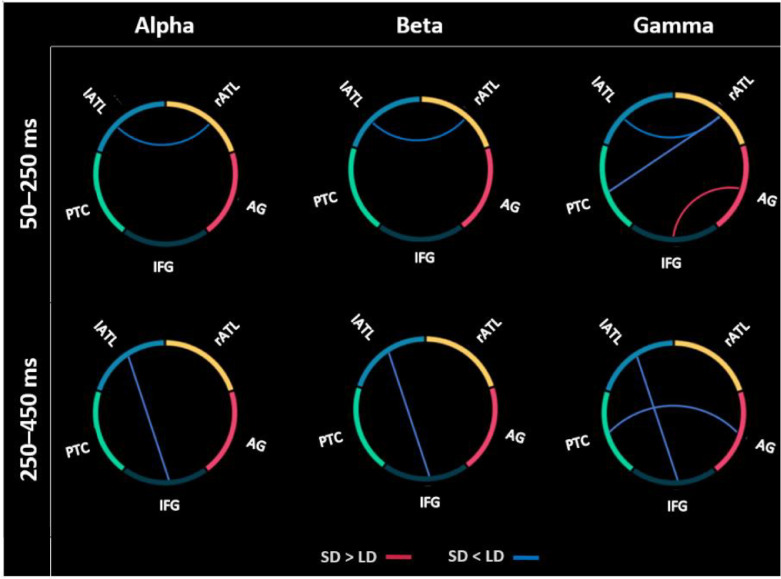
Significant differences in connectivity in the semantic and lexical decision tasks between the semantic ROIs. In the early time window, connectivity between left and right ATL was modulated, and in the later time window the connectivity between lATL and IFG was modulated. Blue and red lines show greater coherence for LD and SD, respectively.

As with the evoked analysis, we sought to corroborate our whole-cortex seed-based analysis using an ROI approach. The paired *t*-test results displayed in Fig. 8 confirm significant task-dependant connectivity between lATL and rATL for alpha (p-value<0.01) and beta (p-value<0.01) bands in the early latency window, as well as between lATL and IFG for alpha (p-value<0.05) and beta (p-value<0.05) bands in the late window. The gamma band also showed task modulation of connectivity between lATL and rATL (p-value<0.05) and between rATL and PTC (p-value<0.05) in the early window, and between lATL and IFG (p-value<0.05) in the late window. Furthermore, the gamma band produced significant connectivity differences between AG and IFG (p-value<0.05), which is the only case where coherence values are larger in the semantic compared to the lexical decision task. In the late window, the gamma band also showed a connection between AG and PTC (p-value<0.05).

This analysis confirmed the early task differences in the connectivity between left and right ATL and the later differences in their connectivity with IFG, found in the whole-cortex seed-based analysis. Pushing the semantic system modulates the connectivity between core regions of the semantic network, especially for the left and right ATL and the IFG.

## 4 Discussion

Semantic cognition critically depends on interactions across a distributed network, yet few studies have elucidated the connectivity of this semantic network using high temporal resolution techniques. Here, we investigated the effects of increasing semantic task demands on spatiotemporal brain activity and functional connectivity in the semantic network estimated from combined EEG/MEG data. We asked how the need for greater semantic cognition modulates responses in the semantic brain network over time, how it affects connectivity amongst putative semantic representation and control regions, and specifically how it modulates the laterality and connectivity of left and right ATL. In our whole-cortex evoked analysis, we observed task differences in bilateral posterior brain regions already present within the 50–150 ms time window, with greater semantic demand resulting in larger activation across much of the semantic network until 350 ms. Early task differences involved inferior parietal and temporal brain regions, which spread further into anterior temporal and parietal regions in both hemispheres between 150 and 250 ms. After 250 ms, the task-modulated evoked activation became left-lateralised and also spread to left inferior frontal regions. Laterality effects in ATL were driven by larger activation in left ATL in the more semantically demanding task. Regardless of the precise assessment method used (whole-cortex seed-based or ROI-based functional connectivity analyses), functional connectivity was modulated by task in multiple frequency bands and time windows, especially between left and right ATL at early latencies in the alpha and beta bands, and between left and right ATL and IFG in later time windows in the alpha and gamma bands. These effects reflected larger desynchronisation in SD compared to LD. Our results indicate that semantic representation and control processes dynamically interact within the first few hundred milliseconds of written word processing, and confirm that the ATL has a central role in the semantic brain network.

Spatiotemporal evidence for the interplay of representation and control processes in dynamic semantic brain networks is still scarce. Here, we contrasted a more semantically demanding (semantic decision) task with a less semantically demanding (lexical decision) task on the same set of well-matched word stimuli. This task contrast does not allow us to unambiguously disentangle representation and control processes, but provides critical information as to the dynamics of the semantic network overall, including the interaction between putative semantic representation and control regions. In addition, the temporal and spectral information presented provides novel insights that will be the basis for future studies on this issue. Importantly, our study included some methodological advancements with respect to the majority of previous EEG/MEG studies on semantic word processing. First, we used combined EEG and MEG recordings to optimise spatial resolution for source estimation using individual realistic head modelling (Hauk et al., 2019; Molins et al., 2008). Second, we present both conventional evoked responses, as well as functional connectivity results in source space in the same study. Third, we provide both whole-cortex and ROI-based results to strike a trade-off between sensitivity and spatial specificity. Fourth, we explicitly evaluated the spatial resolution (“leakage”) of our ROIs as a basis for a critical interpretation of our source estimation results (Hauk et al., 2019). We hope that it will become standard in the EEG/MEG literature to report the relevant leakage indices (or similarly informative measures) in the future.

The role of activity in different frequency bands and possibly “oscillations” in semantic word processing is still unclear, although spectral synchrony (and in particular coherence) has been suggested to be a fundamental physiological mechanism supporting cognition (Farahibozorg et al., 2019; Fries, 2015; Siegel et al., 2012). Thus, in our study we looked at several separate frequency bands to study functional connectivity between pairs of ROIs using coherence. Whilst our evoked analysis showed the expected pattern of more activation for the more semantically demanding task, the opposite seemed to be the case in our functional connectivity analyses, where LD showed larger coherence values compared to SD task in alpha, beta, and gamma bands, from 8 to 36 Hz, but not in the theta band (4–8 Hz). However, coherence values reflect the variability of amplitude and phase across trials (Bastos and Schoffelen, 2016; Lachaux et al., 1999). The LD task is less demanding, resulting in lower reaction times and standard deviations, and presumably lower variability across trials, therefore possibly resulting in larger coherence values. This is consistent with previous findings of desynchronisation across this frequency range (Barca et al., 2011; Cornelissen et al., 2009; Ihara et al., 2003; Klein et al., 2015; Mollo et al., 2017; Wheat et al., 2010). Our explanation is related to the ideas of Hanslmayr et al. (2012) who linked neural within-region desynchronisation to information theory, suggesting that more processing demands can result in larger desynchronization within neuronal populations. Similarly, we argue here that amplitude and synchronization across ROIs are independent, and that larger amplitude but lower coherence in SD both reflect the higher complexity of this task. Therefore, we conclude that our coherence effects reflect task modulation of functional connectivity in the semantic brain network. Our main finding is that ATL emerged as the most dominant region across our different analyses, in the sense that it consistently showed significant effects in multiple latency (especially early) ranges as well as multiple frequency ranges in our functional connectivity analysis. A more detailed analysis of differences amongst different frequency ranges is beyond the scope of this paper. The fact that we found connectivity modulations in alpha, beta and gamma bands may well indicate that they reflect different aspects of more broadband activity in a wider network. This should be investigated in future studies.

Large task modulations were found throughout the putative semantic network, i.e. in bilateral ATLs, IFG, PTC and visual cortices. Engagement of the semantic network is not all or nothing; the information accessed and employed depends upon task demands, even in early word processing (Chen et al., 2015; Chen et al., 2013; Jackson, 2021; Jefferies, 2013; Strijkers et al., 2015). We found early task modulation in visual and inferior parietal areas followed by temporal lobe structures, in particular left anterior temporal lobe and posterior temporal cortex, and then inferior frontal regions. These regions are critical for demanding semantic cognition and are recruited flexibly based on task demands. In particular, the connectivity between left and right ATL and between ATL and IFG supports demanding semantic cognition. This is highly compatible with prior functional connectivity assessments of the semantic network, including the compensatory effects of connectivity between left and right ATLs after transcranial magnetic stimulation (Chiou et al., 2018; Chiou and Lambon Ralph, 2019; Farahibozorg et al., 2019; Jackson et al., 2016; Jung and Ralph, 2019). The selection of task-relevant, and inhibition of task-irrelevant, semantic information is hypothesised to require the interaction of control regions (which represent the task context, including IFG) and representation areas (where task-independent semantic representations are stored, hypothesised to rely principally on the ATLs, Jackson, 2021; Jefferies, 2013; Lambon Ralph et al., 2016). Thus, the differential connectivity between the ATLs and the IFG in the semantically demanding task may reflect the additional interaction required to access the specific subset of features required to answer the difficult semantic decisions.

Very early changes were identified in visual and parietal regions. As described before, the spatial resolution of EEG/MEG does not allow an interpretation at the same level of spatial detail as for fMRI, e.g., with respect to the exact Brodmann areas. This was confirmed by our leakage analysis. However, the high temporal resolution of EEG/MEG allows us to conclude that task modulations that occurred in “early visual areas” (as they are sometimes called in the fMRI literature without timing evidence (e.g. Basti et al., 2019; Mur et al., 2012)) indeed reflect early brain processing, rather than recurrent activation flow (e.g. Lamme and Roelfsema, 2000).

Early task modulation effects were also identified in the AG, a region with a debated role in semantic cognition (Binder et al., 2009; Humphreys et al., 2015; Noonan et al., 2013). Whilst our leakage analysis suggests that AG effects are unlikely due to leakage from PVA, the point-spread and cross-talk functions in Fig. 3 indicate that there could be significant leakage from higher level visual areas posterior to AG but anterior to PVA. Nevertheless, some previous MEG studies have reported AG involvement in semantic processes (e.g., Lewis et al., 2015; Williams et al., 2017). However, in our connectivity analysis, AG does not show rich connectivity with other semantic areas, especially not in the temporal lobes. Additionally, these effects are very early, in parallel with visual areas and prior to any other semantic region. Our ROI-based connectivity analysis (Fig. 8) revealed connectivity modulation between AG and IFG in the early time window, although this was in the opposite direction to all other connectivity differences. Some previous neuroimaging studies have suggested that AG may serve semantic representation (Binder et al., 2009) or control functions (Noonan et al., 2013, but see Jackson 2021), although these assessments are plagued by questions of how to interpret differences in the context of difficulty-dependant deactivation in this region (Humphreys et al., 2015; Humphreys and Lambon Ralph, 2014). Indeed, the AG is consistently found as part of the default mode network (Buckner and DiNicola, 2019) and may play a role in attentional processes. For instance, our early task effects in AG could reflect a change from readiness during rest to the engagement of task networks by this area, resulting in an early increase or “boost” of attentional resources towards the visual word form or the semantic network. Alternatively, a similar function could be achieved by task-positive inferior parietal regions (Duncan, 2010) and the current effects misattributed to the AG region. This hypothesis can be tested in future studies using more fine-grained experimental paradigms.

Our evoked analysis revealed task modulation in ATL starting prior to 200 ms. Previous EEG, MEG and behavioural studies have suggested that semantic information becomes available in visual word processing around this latency (Amsel et al., 2013; Hauk et al., 2012; Pulvermüller et al., 2009), and some EEG/MEG studies have reported activity in ATL regions (Bemis and Pylkkänen, 2013; Dhond et al., 2007; Farahibozorg et al., 2019; Marinkovic et al., 2014; Mollo et al., 2017; Westerlund and Pylkkänen, 2014). This task effect was clearly left-lateralised in our evoked data, which is consistent with findings from neuropsychological and neuroimaging literature that left ATL shows a preference for linguistic stimuli and tasks (Rice et al., 2015a, 2015b). However, connectivity between the ATLs was significant in this early time window, highlighting the possibility of a critical role for the right ATL. Our results also indicate that areas that do not show a significant activity effect can still be part of a distributed network. Indeed, the laterality of the evoked responses changed over time suggesting an interpretation of the necessity of a single ATL may be an oversimplification of a dynamic, recurrent system. This significant functional connectivity in three frequency bands (alpha, beta and gamma) demonstrated that evoked and spectral responses carry independent information.

The connectivity between ATLs and the IFG also varied across time, with significant effects in the later time window. The PTC, another putative semantic control region, was engaged both at a similar time to the IFG (around 300 ms) and at an earlier time point (around 200 ms, with the ATL response). The relative timings of the putative semantic control and representation regions are informative as to their possible interactions. To date, it has been hard to separate the role of IFG and PTC (Jackson, 2021; Jefferies, 2013; Lambon Ralph et al., 2016) and their differential timings could be informative; e.g. could PTC be involved earlier? However, we cannot rule out the possibility that we cannot distinguish the control-related PTC changes from nearby regions engaged in semantic representation, due to the nature of the task manipulation. Perhaps the responses at the two different time points reflect these different elements of semantic cognition, with an early sweep through PTC before the semantic control regions are active. Indeed, although PTC demonstrated task modulated evoked responses, no clear changes in connectivity were identified. This could be a result of the particular connectivity measure chosen, or due to high levels of connectivity with other semantic regions across both tasks.

In conclusion, our results suggest that semantic task demands modulate visual word processing before 100 ms in posterior visual (and perhaps attentional) areas, followed by modulation of multimodal semantic regions; first ATLs and PTC, and then IFG, allowing the context-appropriate extraction of task-relevant semantic features critical for response selection. Our conclusions required the high temporal and reasonable spatial resolution of combined EEG and MEG measurements, as well as the combination of evoked and functional connectivity analyses. Our results raised several questions about the precise mechanisms of the interaction of semantic control and representation, and provide a valuable base to address them in future EEG/MEG studies. In particular, the spatiotemporal resolution of combined EEG/MEG recordings together with sophisticated multivariate and multi-dimensional connectivity methods will be required to characterise dynamic semantic brain networks in more detail (Anzellotti and Coutanche, 2018; Basti et al., 2020, 2019; Kietzmann et al., 2019).

## Declaration of Competing Interest

The authors declare no conflicts of interest.

## Footnotes

1 A previous version of our manuscript has been published as a pre-print: [https://biorxiv.org/cgi/content/short/2021.06.28.450126v1]

2 https://mne.tools/stable/generated/mne.stats.fdr_correction.html

3 https://mne.tools/stable/generated/mne.stats.bonferroni_correction.html
